# Nephroprotection by Hypoglycemic Agents: Do We Have Supporting Data?

**DOI:** 10.3390/jcm4101866

**Published:** 2015-10-23

**Authors:** Jose Luis Górriz, Javier Nieto, Juan F. Navarro-González, Pablo Molina, Alberto Martínez-Castelao, Luis M. Pallardó

**Affiliations:** 1 Hospital Universitario Dr Peset, Universidad de Valencia, Valencia 46017, Spain; E-Mails: molina_pab@gva.es (P.M.); pallardo_lmi@gva.es (L.M.P.); 2 Hospital General Universitario de Ciudad Real, 13005 Ciudad Real, Spain; E-Mail: ljnietoi@gmail.com; 3 Hospital Universitario N S Candelaria, Tenerife 38010, Spain; E-Mail: jnavgon@gobiernodecanarias.org; 4 Hospital Universitario Bellvitge, IDIBELL, Barcelona 08907, Spain; E-Mail: albertomcastelao@gmail.com; 5 GEENDIAB, Diabetic Nephropathy Working Group of the Spanish Society of Nephrology, Santa Cruz de Tenerife 38010, Spain; 6 Carlos III Research Institute, Madrid 28029, Spain

**Keywords:** diabetes mellitus, diabetic nephropathy, diabetic chronic kidney disease, albuminuria, nephroprotection, antihyperglycemic drugs, SGLT2 inhibition, albuminuria, DDP4 inhibitors, glucagon-like peptide agonists

## Abstract

Current therapy directed at delaying the progression of diabetic nephropathy includes intensive glycemic and optimal blood pressure control, renin angiotensin-aldosterone system blockade and multifactorial intervention. However, the renal protection provided by these therapeutic modalities is incomplete. There is a scarcity of studies analysing the nephroprotective effect of antihyperglycaemic drugs beyond their glucose lowering effect and improved glycaemic control on the prevention and progression of diabetic nephropathy. This article analyzes the exisiting data about older and newer drugs as well as the mechanisms associated with hypoglycemic drugs, apart from their well known blood glucose lowering effect, in the prevention and progression of diabetic nephropathy. Most of them have been tested in humans, but with varying degrees of success. Although experimental data about most of antihyperglycemic drugs has shown a beneficial effect in kidney parameters, there is a lack of clinical trials that clearly prove these beneficial effects. The key question, however, is whether antihyperglycemic drugs are able to improve renal end-points beyond their antihyperglycemic effect. Existing experimental data are post hoc studies from clinical trials, and supportive of the potential renal-protective role of some of them, especially in the cases of dipeptidyl peptidase-4 inhibitors, glucagon-like peptide-1 receptor agonists and sodium-glucose cotransporter 2 inhibitors. Dedicated and adequately powered renal trials with renal outcomes are neccessary to assess the nephrotection of antihyperglycaemic drugs beyond the control of hyperglycaemia.

## 1. Diabetes Mellitus and Nephroprotection

Diabetes mellitus (DM) is the leading cause of end stage renal disease in the world. According to the latest figures from the International Diabetes Federation, 382 million people live with diabetes around the world [1] and approximately one in three of them will eventually develop chronic kidney disease [2].

Current therapy directed at delaying the progression of diabetic nephropathy includes intensive glycemic and optimal blood pressure control, proteinuria and albuminuria reduction, interruption of the renin angiotensin-aldosterone system through the use of angiotensin converting enzyme inhibitors and angiotensin type-1 receptor blockers, along with dietary modification and cholesterol-lowering agents [3], including also multifactorial intervention [4]. However, the renal protection provided by these therapeutic modalities is incomplete.

The exact pathogenesis of diabetic nephropathy (DN) is multifactorial, complex and not completely understood. Evidence of that is that an increasing number of studies have indicated that certain diabetic patients do not present the same evolution as was then defined: for example, some often have significant initial deterioration of glomerular filtration rate whereas, in others, microalbuminuria is reduced spontaneously. Chronic kidney disease may be accompanied, rather than preceded, by macroalbuminuria, or it may develop in patients with microalbuminuria or even in those with albuminuria levels that revert to normal. In fact diabetic kidney disease without proteinuria is increasingly recognized [5].

The classic term “diabetic nephropathy” has shifted to a new one “diabetic chronic kidney disease” (DCKD) [6]. From here onwards, this will be the term used in this article. In the diabetic milieu, metabolic derangements and hemodynamic alterations, particularly activation of the renin-angiotensin system, triggers a number of cell signalling cascades, which mediate a cellular response through activation of key transcription factors. In response to such signals, renal cells such as tubular epithelial cells, podocytes, and mesangial cells can produce chemokines, growth factors, and profibrotic cytokines. These responses contribute to a cycle of inflammation, oxidative stress, cellular injury, progressive fibrosis, and loss of glomerular filtration rate. Podocyte loss, endothelial dysfunction, alterations in the glomerular basal membrane and tubular injury contribute to increasing proteinuria during the development and progression of DCKD nephropathy [7].

Intensive control of glycemia and of blood pressure are effective in both preventing the onset and reducing the progression of albuminuria and DN. Antihypertensive agents differ significantly in their albuminuria-lowering capacity despite having similar blood pressure lowering potency. Renin-angiotensin-aldosterone system (RAAS) inhibitors have been shown to be more effective than other drugs. Multifactorial intervention has also been shown to be effective and intensive treatment has demonstrated a 61% reduction in the risk of developing macroalbuminuria and a 55% reduction in the cardiovascular composite end point when the intensive treatment group is compared to the conventional care group [8]. However, little information is available regarding the ability of anti-hyperglycemia agents to lower albuminuria or prevent or slow down the progression of DCKD.

This article analyzes the exisiting data about older and newer drugs as well as the mechanisms associated with hypoglycemic drugs, apart from their well-known blood glucose lowering effect, in the prevention and progression of diabetic nephropathy. Most of them have been tested in humans, but with varying degrees of success. Although some of them have shown promising results, in most cases clinical trials in humans are lacking or have resulted in failure (see summary in Table 1).

## 2. Older Hypoglycemic Treatments and Nephroprotection

### 2.1. Insulin Treatment and Nephroprotection

The benefits of intensive glycemic control were not limited to delaying the onset and slowing the progression of DCKD but extended to decreasing the incidence of cardiovascular diseases, the main cause of mortality in these patients [9]. However, these benefits are related to better blood glucose control, and not related to any specific class of hypoglycemic treatment.

The Diabetes Control and Complications Trial (DCCT) was a milestone in delaying the onset and slowing up the progression of DN in patients with type 1 DM [10]. The United Kingdom Prospective Diabetes Study (UKPDS) showed that intensive blood glucose control by either sulphonylureas or insulin reduced the risk of microvascular complications 22 [11]. And more recent randomized controlled studies in patients with type 2 DM have yielded mixed results. The ADVANCE (Action in Diabetes and Vascular Disease) [12] trial showed that intensive glycemic control reduced albuminuria, nephropathy and the need for dialysis. Likewise, the ACCORD (Action to Control Cardiovascular Risk in Diabetes) trial showed significantly lower rates of albuminuria (but not of more advanced nephropathy) in the intensive glycemic therapy group [13]. Contrary, the VADT (Veterans Affair Diabetic Trial) did not show improvements in either nephropathy or retinopathy with intensive glycemic control [14].

Pilz *et al.* [15] analyzing 1415 healthy, non-diabetic participants demontrated that reduced insulin sensitivity, measured by a hyperinsulinemic–euglycemic clamp, is continuously associated with a greater risk of increasing albuminuria. However, it remains to be clarified whether insulin resistance and albuminuria emerge in parallel as a consequence of a common pathogenic pathway (e.g., endothelial dysfunction) or whether insulin resistance is a causal factor for the pathogenesis of albuminuria. The question of whether insulin treatment is associated with improvements in urinary albumin excretion independently of blood glucose levels must also be clarified.

To sum up, intensive glycemic treatment in both type 1 and type 2 DM reduces the risk and progression of early DCKD, but no data exists as to the specific nephroprotective properties of insulin therapy apart from the benefits derived from blood glucose control.

### 2.2. Metformin

Metformin is a biguanide drug that improves sensitivity to insulin, increases insulin-stimulated uptake and utilization of glucose, reduces basal hepatic glucose production, causes weight reduction and decreases hunger. It is a well-established drug for the treatment of type 2 DM and there are more than 40 million patient years of experience in the past 50 years [16]. Evidence suggests that metformin reduces mortality and morbidity in type 2 diabetes patients. It possesses a cardioprotective property that is independent of its hypoglycaemic effect, and not presented by sulphonylureas or insulin [17]. Additionally, a systematic review of diabetic patients with heart failure has demonstrated that a greater reduction in mortality and hospital admissions is associated with metformin than with any of the other anti-diabetic drugs [18].

In addition to the cardiovascular benefit, some experimental studies have also suggested that metformin has nephroprotective properties. Metformin, in rats, reduces vascular dysfunction and protects against tubular injury induced by gentamicin [19], by modulating oxidative stress on the tubules and restoring biochemical alterations [20,21]. In a diabetic rat model, metformin also protects against tubular cell injury induced by glycosuria, and prevents podocyte injury [22].

Despite these cardiovascular benefits of metformin and the experimental data about nephroprotection, no study has shown any benefit with the use of metformin for renal end points in patients with diabetes mellitus.

### 2.3. Sulfonylureas

There is a lack of studies about the renal effects of sulphonylureas on DCKD. Very recently, an experimental study has investigated the effect of implanting micro-osmotic pumps containing gliquidone into the abdominal cavities of rats with DN [23]. When compared to control or to rats treated with insulin, these authors detected that gliquidone treatment effectively reduced urinary protein. This reducion in proteinuria is probably related to the findings detected in the animals, such as improvement in histological glomerular lesions, promotion of tubular reabsorption and improvement in some biomarkers such as protein kinase C, protein kinase A and tubular expression of megalin and cubilin. The authors suggested that the beneficial effect may be due to a decreased expression of the following receptors: receptors for advanced glycation end products, protein kinase C-β and protein kinase A as well as the tubular expression of the albumin reabsorption-associated proteins (megalin and cubilin) after gliquidone treatment.

A great limitation of the study is the fact that administration of gliquidone via a micro-osmotic pump probably differs from oral administration in terms of therapeutic efficacy. A higher plasma concentration might be obtained with the use of a micro-osmotic pump. If this is the case, the renal excretion of gliquidone will be higher and the glomerular and tubular effects might be more apparent. As a result, it may be difficult to control diabetic nephropathy and lower urinary protein through oral administration of gliquidone.

Another study compared the effect of rosiglitazone, metformin, or glyburide in 4351 recently diagnosed type 2 diabetic patients followed up over 5 years [24]. Only rosiglitazone decreased both albuminuria and diastolic blood pressure. Glyburide had no effect on renal function decline.

Whether these effects would have translated into microvascular and, perhaps more importantly, macrovascular protection is unknown, since the study was not designed to assess these long-term outcomes.

In the Action in Diabetes and Vascular Disease: Preterax and Diamicron MR Controlled Evaluation (ADVANCE) study, a combined approach of routine blood pressure lowering and intensive glucose control resulted in substantial reductions in major renal events and all-cause death [25]. The absence of interaction between blood pressure lowering and intensive glucose control indicates that the effects of these two interventions were independent. However, the study was unable to demonstrate the benefit of glicazide on renal events separately from the effects derived from intensive glucose control.

No other studies have analyzed the effect of sulphonylureas on the development or progression of DN.

### 2.4. Alpha-Glucosidase Inhibitors

Alpha-glucosidase inhibitors such as acarbose slow down carbohydrate digestion and primarily reduce postprandial hyperglycemia, thereby offering an additional therapeutic approach in the long-term treatment of type 2 diabetes.

Although there have been no reported direct effects on DN associated with acarbose treatment, extrapolating data from experimental animal studies suggests that this drug can favourably influence cardiovascular risk factors. Acarbose treatment up-regulates glucagon-like peptide-1 (GLP-1) production, insulin-like growth factor-I (IGF-I) and modulates fibroblast growth factor [26]. Nevertheless, these findings have not been demonstrated in humans.

An analysis from UKPDS studied 1946 type 2 diabetes mellitus patients comparing acarbose *vs.* placebo. After three years, acarbose significantly improved glycemic control irrespective of concomitant therapy for diabetes, with a high percentage of flatulence, 30% *vs.* 12% reported in the placebo group. Urine albumin and insulin sensitivity were not significantly different at any time during the study [27].

### 2.5. Metiglinides

Although two glinides are available, nateglinide and repaglinide, this latter has a more extended use and may be prescribed in patients with differing degrees of renal insufficiency since it is mainly metabolised by the liver. There is a lack of studies analyzing the effect of glinides on albuminuria or the progression of DN.

In order to analyze the effect on hyperfiltration and nitric oxide bioavailability, nine patients newly diagnosed with type 2 DM without microalbuminuria were randomized to a treatment with rosiglitazone or nateglinide, each for 12 weeks [28]. Ten healthy controls were used as placebo group.

Rosiglitazone ameliorated glomerular hyperfiltration in early type 2 diabetes and improved nitric oxide bioavailability. No other renal parameters were analized when nateglinide and rosiglitazone were compared. No other studies have tested the effect of glinides on DN.

### 2.6. Thiazolidinediones

Thiazolidinediones are oral antidiabetic compounds that decrease insulin resistance and stimulate the peroxisome proliferator-activated receptor (PPAR-γ), a nuclear receptor present in various tissues. Apart from improving glycemic control, many background and clinical studies have shown that thiazolidinediones have beneficial effects on other components of metabolic syndrome and cardiovascular risk factors. Moreover, accumulating evidence suggests that these agents may have renal benefits.

Animal studies show that thiazolidinediones decrease urine protein excretion and prevent glomerulosclerosis and tubulointerstitial fibrosis [29,30] and restore podocyte integrity [31]. At the same time several systemic and local renal actions of thiazolidinediones observed in experimental studies may represent plausible mechanisms for these renoprotective properties [32].

A number of human studies have also examined the renal effects of thiazolidinediones on patients with diabetes. Some of them reported significant decreases in urine albumin excretion [33,34,35], whereas others did not show a significant effect [36]. Most of these studies have included a small number of patients.

A meta-analysis that includes 15 studies (five with rosiglitazone and 10 with pioglitazone) involving 2860 patients showed that treatment with thiazolidinediones significantly decreases urinary albumin and protein excretion in patients with diabetes [37].

Nevertheless, the discrepancy between the conflicting results in clinical studies could perhaps be explained by the fact that the risk of albuminuria among patients with type 2 DM, seems to be modulated by, among other factors, the presence of the PPARG-γ2 P12A polymorphism variant. A reduced risk of albuminuria is significantly associated with this phenomenon [37,38]. This fact can result in a heterogeneous response in urinary albumin excretion after treatment with thiazolidinediones.

Several mechanisms have been described to explain the association of thiazolidinediones with renal benefit including improving insulin sensitivity by inhibiting the release of free fatty acids, inhibition of tumor necrosis factor-α, stimulating the production of several insulin-sensitizing adipokines, including adiponectin and visfatin [34,35,37,38].

The favorable renal effect of thiazolidinediones on type 2 DM is not completely dependent on their insulin-sensitizing action. Metformin produces similar glycemic control but has practically no effect on urinary albumin excretion [32,38].

Finally, it is also worth mentioning that rosiglitazone treatment has been shown to cause severe side effects, such as weight gain, fluid retention, and increased cardiovascular risk and it has been withdawn from the market [39]. Pioglitazone is associated with a significantly lower cardiovascular risk and mortality, but in some cases risk of heart failure is increased [18,40] and other concerns such as a slight increased risk of bladder cancer compared to the general population [41]. However, this concern has not been confirmed in recent meta-analyses [42].

Despite the possible benefits of thiazolidinediones in delaying the progression of DCKD, it is important to determine whether the benefit of using pioglitazone outweighs the risks, especially in patients with chronic kidney disease.

## 3. New Hypoglycemic Treatments and Nephroprotection

### 3.1. Incretin-Based Therapies

Incretin-based therapies in the treatment of patients with type 2 DM include the orally active dipeptidyl peptidase-4 (DPP-4) inhibitors (gliptins) and the injectable glucagon-like peptide-1 (GLP-1) receptor agonists. Both share the same mechanism of action, stimulating insulin secretion and inhibiting glucagon secretion. While gliptins act by inhibiting the enzyme that breaks down GLP-1, thus increasing the level of GLP-1 in the blood stream, GLP-1 receptor (GLP-1R) agonists act directly on the beta cell to stimulate insulin secretion by activating signal transduction when glucose is present. When glucose is not present, this receptor no longer couples to stimulate insulin secretion in order to prevent hypoglycemia. Active GLP-1 is responsible for glucose-dependent insulin secretion, suppression of glucagon secretion and delayed gastric emptying.

The antidiabetic action of DPP-4 inhibitors is mediated by slowing the degradation of endogenous GLP-1. DPP-4 inhibitors are, however, not able to raise the GLP-1 to levels achieved after injection of GLP-1R agonists and, therefore, their hypoglycaemic efficacy is less than that of GLP-1R agonists [43]. However, DPP-4 inhibitors may have therapeutic benefits that extend beyond glucose lowering. Data suggests that GLP-1 has direct renal and cardiac effects, and that the GLP-1R is localized in extra-pancreatic tissues, including the kidney and heart. DPP-4 inhibitors block the degradation of endogenous GLP-1 and might also influence circulating levels and activity of other vasoactive peptides that could act on the kidney [44,45].

GLP-1R agonists also have effects beyond glucose control that may be of indirect help for nephroprotection: they reduce body weight, induce satiety and influence the gastrointestinal system (gut motility, pancreatic exocrine enzyme secretion) and the cardiovascular system (endothelial and myocardial function). GLP-1R agonists reduce body weight, whereas DPP-4 inhibitors do not affect body weight [46]. In general DPP4 inhibitors ameliorate kidney disease to a lesser extent than do GLP1R, by improving weight-related risk factors including body fat content and distribution [46].

Evidence from animal and human studies indicates that incretin-based therapies might prevent the onset and progression of DCKD, as measured by clinical and histological improvements. Incretin-based therapies may positively influence haemodynamic variables (hyperfiltration, glomerular capillary hydraulic pressure, and systemic blood pressure), metabolic factors (glycaemia, dyslipidaemia, oxidative stress) and inflammatory pathways in the pathogenesis of DCKD [46].

In addition, there is some evidence that GLP-1R agonists and DPP-4 inhibitors mediate sodium excretion and diuresis to lower blood pressure. The pleiotropic actions of DPP-4 inhibitors are ascribed primarily to their effects on GLP-1 signalling, but other substrates of DPP-4, such as brain natriuretic peptide and stromal-derived factor-1a, may play other roles [47]. Collectively, these pleiotropic effects may reduce the risk of DCKD in patients with type 2 DM [47]. The beneficial effects of incretin-based therapies on renal risk factors in type 2 DM seem to go beyond glucose control and might thereby confer renoprotection.

In the human kidney, expression of GLP‑1R mRNA was demonstrated by *in situ* hybridization, and GLP-1R expression was detected using ^125^I-labelled GLP-1 [48] but the exact cellular location of GLP-1R in the kidney, both in humans and animal species, requires further study.

#### 3.1.1. Dipeptidyl Peptidase-4 Inhibitors

DPP-4 inhibitors (gliptins) are emerging as second line treatment ahead of sulphonylureas or metformin due to a possible beneficial effect on the beta cell and their weight neutrality. They are effective, well tolerated and have been shown to be safe in general. All of them, except for linaglitin, require dose adjustment based on glomerular filtration rate in order to avoid plasma accumulation and side effects.

The main action of DPP-4 inhibitors is to increase the levels of endogenous incretin hormones, especially GLP-1. But DPP-4 inhibitors are also bound to the surface of many cell types including kidney proximal tubular cells and endothelial cells [49]. Microvesicle-bound DPP-4 is secreted from tubular epithelial cells. It is found in urine and may be an early marker of renal damage before the onset of albuminuria [49].

##### 3.1.1.1. Experimental Data

Sun *et al.* [49] also described higher urinary microvesicle DPP-4 levels in patients with DM compared to non-diabetic controls that positively correlated with the extent of albuminuria in patients. Upregulation of DPP-4 expression in renal glomeruli occurs during inflammation and usually accompanies the development of diabetes-induced glomerulosclerosis [50].

Renal effects of DPP-4 inhibitors appear to be, at least in part, mediated by increased GLP-1 levels [51]. In addition to the pancreas, GLP-1R protein is expressed predominantly in proximal tubules [52] and numerous other tissues including glomerular endothelial cells, mesangial cells and podocytes. Its expression was decreased in diabetic compared with nondiabetic mice [51].

In the kidney, DPP-4 is expressed on the brush border of proximal tubules and glomerular podocytes [53]. DPP-4 is also excreted in the urine.

Preclinical data suggesting the nephroprotective effects of DPP- 4 inhibitors can de attributed to sitagliptin [54], vildagliptin [55], and linagliptin [56]. Treatment with DPP-4 inhibitors in diabetic rats lowered glycemia and ameliorated glomerular, tubulointerstitial, and vascular lesions. It also reduced kidney lipid peroxidation as measured by decreased malondialdehyde content. Light and electron microscopic studies of renal tissue revealed inhibited interstitial expansion, glomerulosclerosis, and thickening of the glomerular basement membrane [55].

However, since the discovery of DPP-4 as an adenosine deaminase binding protein [57], the expression of DPP-4 has been considered a marker of renal injury, including diabetic nephropathy. Several studies have demonstrated the nephroprotective effects of DPP-4 inhibitors on experimental animals [58].

Liu *et al.* [55], using a type 1 DM model, minimized the effect of DPP-4 inhibition on insulin release as streptozotocin destroys the islet cells in the pancreas. As a result, it is thought that the glucose-lowering effect of the incretin system is nullified. In that study the authors reported that vildagliptin was renoprotective, with a reduction in albuminuria and improvement in the histological changes in the kidney, associated with reduced DPP-4 activity and increased GLP-1 levels. The authors concluded that these changes were probably not attributable to the hypoglycaemic effect of vildagliptin.

In streptozotocin treated rats, when compared to vehicle, treatment with linagliptin has shown reduced glomerulosclerosis after treatment with linagliptin alone or in combination with angiotensin receptor blockade [56].

Sitagliptin ameliorated renal lesions, including glomerular, tubulointerstitial, and vascular lesions [56,59,60,61], in Zucker diabetic rats. Whether these effects were direct or dependent on glucose reduction was not ascertained. Nevertheless, another study reported that sitagliptin decreased IL-1β, TNF-α levels and Bid protein levels [60], indicating protective effects against inflammation and apoptosis in the kidney. Another study has shown that linagliptin reduced AGE and RAGE levels and oxidative stress, improved albuminuria, and ameliorated the histological features of glomerulopathy [62].

A more recent study by Vavrinec *et al.* [63] showed that vildagliptin, without affecting plasma glucose levels or proteinuria, was able to decrease glomerulosclerosis and restore myogenic arteriolar constriction to normal levels, possibly due to reduced oxidative stress. This series of experimental studies shows that DPP4-inhibitors at the kidney level may promote both negative and positive effects, with most data pointing to the protective effects of DPP4-inhibitors on kidney function.

Whether these effects are direct or partially mediated by changing glucose concentration warrants further scrutiny, even though results obtained in models of Type 1 DM seem to support a direct effect.

Besides GLP-1 and GIP, DPP-4 cleaves multiple substrates, such as brain natriuretic peptide (BNP), ANP, substance P, among others, many of which are vasoactive [64]. On the other hand, DPP-4 is also expressed at the apical brush border surface of renal proximal tubular cells and also has GLP-1 independent renal and cardiovascular actions [64].

##### 3.1.1.2. Clinical Data

Few studies have been devoted to directly assessing the effects of DPP4 inhibitors on renal functional measures and the key question is whether DPP4 inhibitors are able to improve renal end-points beyond their antihyperglycemic effect.

In some uncontrolled studies with small number of patients, six months of treatment with sitagliptin [65] for 12 or 17 weeks of treatment with alogliptin [66] reduced albuminuria in patients with type 2 DM. This data was confirmed and expanded in a pooled analysis of phase III trials of linagliptin, which showed a significant reduction in albuminuria after a mean of 24 weeks of treatment. The urinary albumin-to-creatinine ratio was reduced by 32% at week 24 in the linagliptin group compared with 6% in the placebo group. The significant reduction achieved in the albuminuria was already detected in week 12 (−29%) and was independent of blood pressure levels and HbA1c [67]. Interestingly, 16 weeks of treatment with exenatide reduced both albuminuria and urinary levels of TGF-α and type IV collagen (versus glimepiride) in patients with type 2 DM [68]. Of note, the degree of urinary albumin-to-creatinine ratio reduction did not correlate with the level of change in HbA1c, thereby suggesting that the effect of the DPP-4 inhibitor on urinary albumin-to-creatinine ratio may have been independent of the improvement in glycemic control [67].

A similar inhibitory trend against the development and progression of microalbuminuria was observed in another study using saxagliptin in patients with type 2 diabetes and cardiovascular complications [69], although it was unclear whether the observed effect was due to a significant improvement in glycemic control or whether it was associated with a subsequent decrease in cardiovascular or renal complications.

Another phase IIIb, multicenter, randomized, double-blind, placebo-controlled, parallel-group study, evaluating the glycemic and renal efficacy of linagliptin in subjects with type 2 diabetes and renal impairment, is the MARLINA study (Efficacy, Safety & Modification of Albuminuria in Type 2 Diabetes Subjects With Renal Disease With LINAgliptin) (https://clinicaltrials.gov/ct2/show/NCT01792518 Identifier: NCT01792518). It is currently ongoing.

In a pooled analysis from a large clinical trials program of the DPP4 inhibitor linagliptin in type 2 DM, that included 5466 patients, linagliptin was not associated with increased renal risk but was associated with a significant reduction in clinically relevant kidney disease end points in patients with type 2 diabetes [70].

There have been recent concerns about the disconnection between the glucose-lowering effect and cardiovascular safety of certain diabetic agents. After the association between rosiglitazone use and increased cardiovascular risk was reported [71], several trials have been performed to determine the cardiovascular safety of DPP4 inhibitors in diabetic patients with various degrees of renal impairment. Undoubtedly, cardiovascular benefit may affect renal outcomes.

Two such trials for DPP-4 inhibitors have been published [69,72]. These placebo-controlled studies, involving a median 2 years of treatment with alogliptin and saxagliptin, respectively, demonstrated no cardiovascular harm and a modest reduction in albuminuria progression in high-risk patients with type 2 DM, most of whom had a history of cardiovascular disease. *Post hoc* analyses of these trials did not detect any effects of DPP‑4 inhibitors on clinically relevant renal end points. However, this data should be interpreted with caution, as the trials were not adequately powered to study the effects of DPP‑4 inhibitors on renal outcomes. Newer studies will be published shortly (TECOS: Tial Evaluating Cardiovascular Outcomes with Sitagliptin) and medium long-term (CAROLINA: Cardiovascular Outcome Study of Linagliptin Versus Glimepiride in Patients with Type 2 Diabetes) and CARMELINA (linagliptin in cardiovascular an renal outcomes) and also MARLINA trial, studying the effects of linagliptin on microalbuminuria in type-2 DM patients, that will help us to completely assess cardiovascular and renal safety.

#### 3.1.2. Glucagon-Like Peptide-1 Receptor Agonists

##### 3.1.2.1. Experimental Data

Apart from the several effects described above, the effects of GLP-1R agonists and DPP-4 inhibitors on kidney disease have been extensively studied in diabetic kidney disease models, where they may have protective roles in reducing proteinuria and glomerular sclerosis, which are associated with protection from endothelial injury and reduction in oxidative stress and inflammation [47,73]. Of note, these effects of incretins appear to be independent of glycemic control [73,74].

Experimental studies using various diabetic models suggest that incretins protect the vascular endothelium from injury by binding to GLP-1 receptors, thereby ameliorating oxidative stress and the local inflammatory response, which reduces albuminuria and inhibits glomerular sclerosis [47].

Initial studies in hypertension-prone rats showed that GLP-1 prevented the development of hypertension, ameliorated histologically verified renal damage and reduced albuminuria [75].

There is evidence to suggest that long-term treatment with the GLP-1R agonist exendin-4 ameliorates DN in both type 1 and type 2 DM animal models, most probably through its action on the glomerular endothelial and infiltrating inflammatory cells [74].

In humans, GLP-1 promotes natriuresis by acting on the proximal tubule, thereby also increasing the fractional excretion of lithium (as a measure of end-proximal tubule delivery) [76]. GLP-1 also reduces water and salt intake in rats, healthy men, and obese individuals [77].

##### 3.1.2.2. Clinical Data

In the study of Zangh *et al.* [68], 31 type 2 diabetic patients with microalbuminuria were randomly allocated to receive exenatide or glimepiride treatment. At 16 weeks, after correcting for blood pressure, glucose level and body mass index, 24-h urinary albumin, urinary TGF-β1 and type IV collagen were significantly lower in the exenatide group than in the glimepiride group (*p* < 0.01), while glycemic control showed no statistical difference between the two groups. These results suggest that exenatide reduces urinary TGF-β1 and type IV collagen excretion in patients with type 2 diabetes and microalbuminuria, which may be partly contributory to its directly renoprotective role [68].

In phase III trials included in meta-analyses, GLP-1R agonists decreased systolic blood pressure by 2–5 mmHg, having a modest effect on diastolic blood pressure (0.5–2 mmHg), although not achieving statistical significance [78]. The antihypertensive action of GLP-1R agonists might be partly attributable to a combination of GLP-1-mediated natriuresis and diuresis, but could also involve improved endothelial function, increased release of vasoactive factors (such as nitric oxide and atrial natriuretic peptide), a decrease in ET-1 levels and alteration in the balance of the autonomic nervous system [79,80].

GLP-1, GLP-1R agonists and DPP-4 inhibitors also affect renal haemodynamics [81]. The GLP-1-related increase in sodium delivery to the macula densa restores disrupted tubuloglomerular feedback associated with diabetes, resulting in relative vasoconstriction of the afferent renal arteriole and, consequently, a decrease in capillary hydraulic pressure. However, pathways that affect renal haemodynamics independently of tubuloglomerular feedback also seem to be present, suggesting an interaction with vasoactive [43,82].

As occurs with DPP4 inhibitors, cardiovascular safety is a key point with GLP1-R analogues. Large-scale clinical studies with cardiovascular end points are underway, although much needed studies with renal end points are still required.

DPP4 inhibitors have shown neutral effect in cardiovascular outcomes [69,72]. Nevertheless, the first studies published with GLP1-R analogues have shown a positive effect on cardiovascular outcomes. This may have a beneficial effect on renal outcomes. In a large retrospective analysis [83], patients treated with the GLP-1R analogue exenatide were shown to have a 20% reduction in cardiovascular events in comparison with other glucose-lowering agents. To a certain extent, improvements in surrogate cardiac outcomes observed in GLP-1 or GLP-1 analogues have been found to be independent of their euglycaemia achievements [84].

The effects of GLP-1 or its analogues on cardiovascular outcomes may be related to a direct effect on cardiomyocytes mediated both by GLP-1-increased myocardial insulin sensitivity and subsequent glucose uptake, endothelial function, endothelial cells and vascular smooth muscle cells and pro-inflammatory markers [85].

To date, mechanistic investigation of their effects on microvascular or large-vessel diabetic vascular disease is lacking. LIRA-RENAL Study is ongoing (The Effect of Liraglutide in Patients With Prediabetes and Kidney Failure). Regardless of the mechanisms involved, ongoing cardiovascular outcome trials including EXSCEL (Exenatide Study of Cardiovascular Event Lowering Trial), LEADER (Liraglutide Effect and Action in Diabetes), and also HARMONY (Effect of Albiglutide, When Added to Standard Blood Glucose Lowering Therapies, on Major Cardiovascular Events in Subjects With Type 2 Diabetes Mellitus) may help to dissipate doubts about cardiovascular safety.

### 3.2. Sodium-Glucose Cotransporter-2 Inhibitors

Sodium-glucose cotransporter-2 (SGLT2) inhibitors are emerging as new therapies with complementary mechanisms of action that are independent of insulin secretion or action. They have acceptable safety profiles and may provide additional therapeutic options to achieve glycaemic control and renoprotection. This renoprotection may be derived from direct effects, such as attenuating diabetes-associated hyperfiltration and tubular hypertrophy, as well as reducing the tubular toxicity of glucose and indirect effects such as improving glycaemic control, reducing insulin levels and improving insulin sensitivity, improving weight control due to modest reductions in body weight, improving blood pressure control due to the natriuretic effect and weight loss and lowering uric acid levels [86].

Like agents that block the renin–angiotensin system, SGLT2 inhibitors also reduce single-nephron glomerular filtration rate in the chronically diseased kidney, though by quite different mechanisms. In case of SGLT2 inhibitors these drugs reduce sodium reabsorption in the proximal tubule, so increasing delivery to the macula densa, augmenting the tubuloglomerular feedback and reducing single-nephron glomerular filtration rate [87,88]. This may be nephroprotective as in diabetic patients hyperglycemia causes increases in proximal tubular reabsorption secondary to induction of tubular growth with associated increases in sodium/glucose co-transport. This increase in proximal reabsorption leads to a decrease in solute load to the macula densa, deactivation of the tubuloglomerular feedback, and an increase in glomerular filtration rate. Glomerular hyperfiltration is currently recognized as a risk progression of kidney disease in diabetic patients. Limiting proximal tubular reabsorption by SGLT2 inhibitors constitutes a potential target to reduce hyperfiltration [89].

These beneficial effects may not be present in patients with established renal impairment. This may be due to the fact that the magnitude of glucose excretion and haemoglobin A1c reduction induced by SGLT2 inhibitors is dependent upon the filtered glucose load and is maximal in diabetic subjects with normal glomerular filtration rate (GFR). However, a high filtered glucose load is associated with only modest glucose reduction in patients with renal impairment, where the filtered glucose load is reduced. As a consequence, in this context, SGLT2 inhibitors show a decrease of glucose lowering efficacy.

Moreover, DCKD is associated with impaired autoregulation of renal blood flow, meaning that drops in blood pressure will be more likely to be associated with reduction in renal perfusion in the diabetic kidney. This is particularly the case in patients with established renal impairment. In some cases, volume depletion and blood pressure lowering associated with SGLT2 inhibitors has been associated with acute-on-chronic renal impairment [90]. This fact should be taken into account given that their effect on blood pressure, natriuresis, osmotic diuresis, volumen depletion and weight loss, hypotension has also been found to be a side effect of SGLT2 inhibition, especially in patients taking loop diuretics [91].

Monitoring of renal function is currently justified when using RAAS blockade in patients with diabetes and similar considerations may also be appropriate in selected (volume sensitive or patients with increased hypertensive response to sodium depletion stimuli) patients receiving SGLT2 inhibitors, especially as nearly all diabetic patients receive RAAS blockade.

#### 3.2.1. Experimental Data

Experimental evidence has been collected to determine whether SGLT2 inhibitors act in a nephroprotective manner in diabetes. Some experimental studies have shown beneficial effects via the different mechanisms by which SGLT2 inhibitors may be associated with nephroprotection. For this purpose experimental models have been set up in rodents. These studies have shown a reduction in the inflammatory response in the kidney, such as less macrophage infiltrates, lower cytokine levels (TGF-β and MCP-1) as well as lower apoptosis rates, and indicate anti-oxidative effects through SGLT2 inhibition. These effects are independent of its effects on blood pressure or glucose control [92].

On the other hand, SGLT2 inhibitors are able to attenuate the hyperfiltration associated with experimental diabetes [93,94]. This is thought to be mediated by the reduction in proximal sodium absorption when SGLT2 is inhibited, leading to increased distal sodium delivery to the macula densa, suppression of tubuloglomerular feedback pathways and a compensatory and persistent reduction in intraglomerular pressure.

Renal angiotensin aldosterone blockade is one of the keys to nephroprotection in diabetic kidney disease. Indeed in one experimental study, the combination of RAAS blockade with SGLT2 inhibition was associated with additive renoprotective effects compared to either drug alone [95].

Finally, the use of SGLT2 inhibitor empaglifozin has demosntrated that inhibition of glucose reabsorption was able to attenuate the renal hypertrophy associated with experimental diabetes [96,97], reduce albuminuria and markers of renal inflammation [98].

#### 3.2.2. Clinical Data

Dedicated renal protection studies have not yet been completed on humans, although such studies are currently underway (Evaluation of the Effects of Canagliflozin on Renal and Cardiovascular Outcomes in Participants with Diabetic Nephropathy (CREDENCE) trial-NCT02065791).

Clinical data on nephroprotection of SGLT2 inhibitors come only from clinical trials, conducted with end points of efficacy and safety, not with renal end points. Few clinical studies examined the effects of SGLT2 inhibition on renoprotection and glycaemic control in CKD [99,100,101,102,103,104].

In these studies the urinary albumin/creatinine ratio fell in treatment groups and increased or decreased at a lesser extent in controls. These studies show results at short and medium term, and glomerular filtration rate showed a slight decrease in eGFR that was observed due to the haemodynamic effect of SGLT2 inhibitors. Nevertheless, after a follow-up greater than 80 weeks, a reverse pattern was observed in a small number of participants. This finding should be confirmed in a larger population using renal measurements as primary end-points. Unfortunately, these large trials do not offer insights into the potential nephroprotective effect of these agents due to the short-term follow-up.

One of the major issues still under debate is the cardiovascular risk profile of SGLT2 inhibition. The incidence of cardiovascular events was observed to increased in the first 30 days post-initiation of treatment probably due to volume depletion and hypotensive episodes [105]. Similarly, stroke may occur more often in patients undergoing hypotensive episodes. Several large, long-term studies with cardiovascular endpoints are ongoing and will provide data in the next two to six years. Those include the DECLARE-TIMI58 for dapagliflozin (expected in 2019), CANVAS for canagliflozin (expected in 2018), and EMPA-REG OUTCOMES. This latter study has been recently published, showing that patients with type 2 diabetes at high risk for cardiovascular events who received empagliflozin, as compared with placebo, had a lower rate of the primary composite cardiovascular outcome and of death from any cause when the study drug was added to standard care. This study supports a strong evidence for a reduction in cardiovascular risk with the use of a SGLT2 inhibitor (empaglifllozin) [106].

Very recently the US Food and Drug Administration (FDA) and the the European Medicines Agency (EMA) have begun a review of SGLT2 inhibitors used to treat type 2 DM to evaluate the risk for diabetic ketoacidosis. This is as a consequence of the appearance of 121 cases of euglycemic asymptomatic diabetic ketoacidosis with blood sugar levels only moderately increased. During the review review, healthcare professionals will be informed about the risk of diabetic ketoacidosis and how to manage it in those patients. More information is needed about this complication as well as its possible relation with the drug. To date there is no data to suggest that this complication can directly affect renal function.

Taken together, these findings suggest that SGLT2 inhibitors may have a renoprotective effect with a decline in albuminuria and a long-term maintenance of glomerular filtration rate. However at present, it is not possible to determine if the above-mentioned effects have a long-term advantageous impact on the progression of diabetic nephropathy. It is also not posible to determine whether the decline in albuminuria is related to indirect effects (blood pressure reduction, increased of natriuresis, weigth loss or improving glycaemic control), changes in intraglomerular hemodynamic and pressure, as demonstrated for ACEi and sartans, or whether it may be ascribed to other actions of SGLT2 inhibitors on renal function. Interestingly, the pattern of an acute reduction followed by a stabilization in GFR reported in these studies on SGLT2 inhibitor groups is similar to those reported with angiotensin converting enzyme inhibitor in subjects with CKD but with different underlying mechanisms for the changes in GFR [99,100,107].

**Table 1:** Summary of renal effects of hypoglycemic drugs on nephroprotection: experimental and clinical studies.

Drug	Direct Renal Nephroprotective Mechanisms	Indirect Nephroprotective Mechanisms (Apart from Glycaemic Control)	Nephroprotective Effect in Clinical Studies	Clinical Trials
**Insulin**	↓insulin sensitivity is associated with ↑risk of albuminuria [15]		Indirect data from UKPDS study	Not done
**Metformin**	Reducing vascular dysfunction and oxidative stress in rats [19,20,21]	Cardiovascular benefit	Not done	Not done
**Sulphonylureas**	↓proteinuria IN animal models: Improvement in histological glomerular lesions, promotion of tubular reabsorption of some biomarkers by↓expression of PKC-β, PKA, megalin and cubilin [23]		No effect on albuminuria [24]	
**α-Glucosidase inhibitors**	Up-regulates GLP-1 production and IGF-1 in experimental models [26]		Not done	Not done
**Metiglinides (repaglinide)**			No differences in albuminuria compared with metformin or insulin [26]	Not done
**Thiazolidinediones**	Improving insulin sensitivity. Inhibition of TNF-α [34] Improving histological lesions, decrease proteinuria and and restore podocyte in animal models [29,30,31]	Improvement of metabolic síndrome and cardiovascular risk factors [29,30]	Heterogeneus response in albuminuria Benefit decreasing albuminuria in meta-analysis Conflicting results Small number of patients [33,34,35,36,37]	Not done
**DPP-4 inhibitors**	Ameliorating histological lesions in rats [55] ↓IL-1β, ↓TNF-α, ↓Bid protein levels in experimental studies [60]		Sitagliptin decreases albuminuria after 6 moths of treatment [65,66,67]	MARLINA trial (on going)
**GLP-1R analogs**	Ameliorated renal histological lesions in animal models [74] Promoting natriuresis acting in proximal tubule in humans [77] restoring tubulo-glomerular feedback [81]	Reducing blood pressure and increasing natriuresis [78]	Exenatide reduces albuminuria and TFG-β1 and type IV collagen excretion and microalbuminuria compared to glimepiride in patients with type 2 diabetes mellitus [68]	Not done
**SGLT2 inhibitors**	-Attenuating diabetes-associated hyperfiltration and tubular hypertrophy (Thomas) -Reducing the tubular toxicity of glucose - Reducing single-nephron glomerular filtration rate - In experimental diabetes reduce albuminuria and markers of renal inflammation - In animal models SGLT2 inhibits inflammatory response in kidney (TGF-β, MCP-1), and ↓apoptosis rates - Restoring tubuloglomerular feedback [92,93,98]	Decreasing weight and blood pressure, improving glycaemic control and increase in sodium excretion	Not done	CREDENCE trial (On going). Indirect data from previous clinical trials

**DPP-4:** dipeptidyl peptidase-4; **GLP-1R:** glucagon-like peptide-1 receptor; **SGLT2:** Sodium-glucose cotransporter-2; References are in brackets.

## 4. Conclusions

In conclusion, SGLT2 inhibitors are a new class of antidiabetic drugs that induce a moderate effect on blood glucose, in chronic kidney disease patients. Existing data are supportive of a potential renal-protective role for SGLT2 inhibition in patients with diabetes. Further studies are needed to investigate whether SGLT2 inhibitors have renoprotective effects beyond the control of hyperglycaemia in diabetic patients as well as subjects with established DCKD. Nevertheless, they may represent a significant additional therapeutic tool to the current trreatments in the clinical prevention and management of diabetic nephropathy. Dedicated, adequately powered, renal outcome trials are eagerly awaited to assess the clinical utility of SGLT2 inhibition as a renal-protective therapy.

Although experimental data about most antihyperglycemic drugs has shown a beneficial effect in kidney parameters, there is a lack of clinical trial results that clearly prove these beneficial effects. The key question, however, is whether antihyperglycemic drugs are able to improve renal end-points beyond their antihyperglycemic effect. Existing experimental data comes from post hoc studies from clinical trials, and are supportive of the potential renal-protective role of some of them, especially dipeptidyl peptidase-4 inhibitors, glucagon-like peptide-1 receptor agonists and sodium-glucose cotransporter 2 inhibitors. Dedicated and adequately powered renal trials with renal outcomes are neccessary to assess the nephroprotection of antihyperglycaemic drugs beyond the control of hyperglycaemia.
